# Unilateral Swollen Hand: A Rare Case of Primary Lymphedema Tarda

**Published:** 2015-09-16

**Authors:** Akinyemi Idowu, Kingyin Lee, Sameer Gujral, Jacob Manushakian

**Affiliations:** Department of Plastic Surgery, Derriford Hospital, Plymouth, United Kingdom

**Keywords:** hand, swelling, lymphedema, lymphoscintigraphy, lymphoscintigram

## DESCRIPTION

An unemployed female of 36 years presented with acute swelling of her right dominant hand without a history of trauma, infection, surgery, or radiotherapy. Hand neurology and blood tests were normal; autoimmune screen was negative. Venous thrombosis was excluded. Lymphoscintigraphy showed no radiolabeled colloid uptake, suggesting a rare diagnosis of unilateral primary lymphedema of the hand.

## QUESTIONS

1. **What is lymphedema and how is it classified?**
2. **What are the causes of lymphedema and the differential diagnoses in this case?**
3. **How is a diagnosis of primary lymphedema made?**
4. **How is lymphedema managed?**

## DISCUSSION

Lymphedema is characterized by fluid retention and tissue swelling caused by an imbalance between lymphatic production and drainage. It commonly results from lymphatic obstruction, leading to accumulation of protein-rich lymph in the interstitium. Swelling caused initially by fluid becomes increasingly due to fibrosis. Clinically, this manifests as edema, becoming increasingly firm and resistant to pitting. This leads to trophic changes in the skin with thickening, loss of elasticity, and propensity for infections, exacerbating the cycle. Lymphedema is classified as primary (can be hereditary) or secondary (acquired). Primary lymphedema is rare, especially when unilateral.^1^ It is categorized by age: congenital lymphedema (at birth), lymphedema praecox (puberty to 35 years of age), and lymphedema tarda (>35 years of age). Secondary lymphedema (SL) occurs following trauma, surgery, radiotherapy, immobility, or infection.

*Congenital lymphedema* presents at birth or onset of ambulation, usually being confined to the legs. *Milroy disease* describes bilateral congenital lymphedema of the legs involving autosomal dominant transmission of mutation in the VEGFR-3 tyrosine kinase gene.^2^ Cases have also been reported in other chromosomal abnormalities, but most are sporadic. *Meige disease* is caused by autosomal dominant defects in the FOX2^3^ gene, resulting in the absence of valves and lymph capillary wall abnormalities, presenting with bilateral pubertal lower limb edema. *Lymphedema praecox*, the commonest primary subtype, is more frequent in females, tending to present at puberty, affecting lower limbs. A variety of mutations have been identified, but most cases are sporadic. *Lymphedema tarda* (rarest of the primary causes) is the least well understood; 85% of these are unilateral. It is uncertain whether it is due to congenital abnormality, causing morbidity later in life or an unknown acquired defect. Lymphedema tarda in the upper limb is uncommon, and documented cases have involved the entire limb.^4^ Unilateral localized hand and wrist lymphedema is even rarer. Secondary lymphedema of the arm is well documented. Commonly, it arises following axillary lymphadenectomy. It is also described in conjunction with rheumatoid and psoriatic arthritis, contact dermatitis, radiation, and trauma.^1^

Differential diagnoses for unilateral limb lymphedema include congenital causes, trauma, neoplasia, vascular anomalies, “factitious” causes, and infection. When causes of SL have been excluded, a diagnosis of primary lymphedema may be made. Lymphoscintigraphy may be useful in confirming abnormalities suggestive of SL. Documented causes of unilateral hand and wrist swelling also include “factitious” lymphedema^1^ and puffy hand syndrome.^5^ Factitious lymphedema is associated with abnormal psychology and a desire for material gain. Animal experiments have shown lymphedema is reproducible using recurrent direct blows to the upper limb.^6^ In intravenous drug use, puffy hand syndrome is recognized because of repeated injections into the hand, characterized by swelling of the hand and forearm, with traumatic disruption of deep lymphatics.^5^ These causes were excluded in this case.

Management goals in lymphedema are to restore function, minimizing physical and psychological morbidity, and to prevent infection and chronic change. In SL, any precipitants such as neoplasm or infection should be treated. Prompt therapy is key to minimize irreversible fibrosclerotic changes within tissues. Few pharmacological treatments have any benefit. Meticulous skin hygiene is vital, treating wounds promptly and cleansing and moisturizing skin. Physical therapies are often first-line therapies, including compression (minimum 40 mm Hg), layered bandaging, physical exercise, massage, pneumatic pumps, and elevation.^1^ Encouraging weight loss, avoiding constrictive clothing that might cause a tourniquet, and avoiding trauma are important. Advances in surgical techniques could reduce or minimize symptoms.^7^ Supermicrosurgical techniques for lymphedema recently used include lymphovenous bypass and vascularized lymph node transfer, but they are not widely available. Best results are seen in patients with early-stage lymphedema.^7^ Active cancer is considered a contraindication to lymphedema surgery.

## Figures and Tables

**Figure 1:**
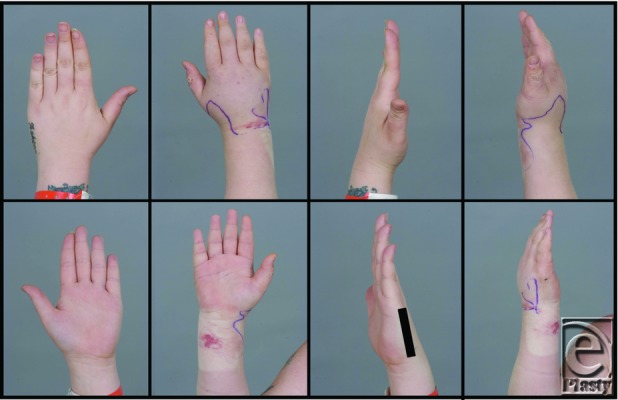
Clinical comparison of the right swollen hand versus the left unaffected hand.

**Figure 2:**
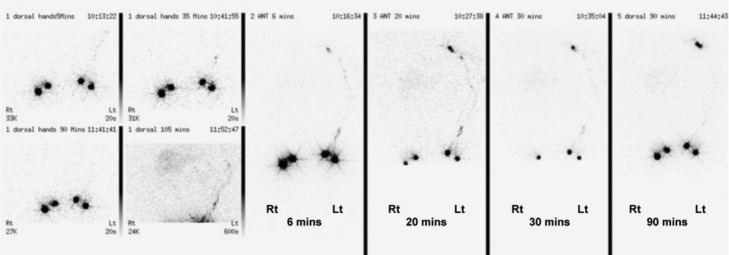
Lymphoscintigram of bilateral upper limbs. The tracer was injected into the web spaces of 2 fingers on both hands with imaging up to 90 minutes postinjection. On the left, axillary nodes are visible at 6 minutes. On the right, the tracer did not seem to leave the hand for up to 90 minutes, consistent with a lymphatic blockage.
